# Validating a double Gaussian source model for small proton fields in a commercial Monte-Carlo dose calculation engine

**DOI:** 10.1016/j.zemedi.2022.11.011

**Published:** 2022-12-26

**Authors:** Fabian Kugel, Jörg Wulff, Christian Bäumer, Martin Janson, Jana Kretschmer, Leonie Brodbek, Carina Behrends, Nico Verbeek, Hui Khee Looe, Björn Poppe, Beate Timmermann

**Affiliations:** a West German Proton Therapy Centre Essen (WPE), Essen, Germany; b University Hospital Essen, Essen, Germany; c West German Cancer Centre (WTZ), Essen, Germany; d Department of Particle Therapy, Essen, Germany; e Faculty of Physics, Heinrich Heine University Düsseldorf, Düsseldorf, Germany; f German Cancer Consortium (DKTK), Heidelberg, Germany; g Department of Physics, TU Dortmund University, Dortmund, Germany; h RaySearch Laboratories, Stockholm, Sweden; i University Clinic for Medical Radiation Physics, Medical Campus Pius Hospital, Carl-von-Ossietzky University, Oldenburg, Germany; j Department of Radiation Oncology, University Medical Center Groningen, University of Groningen, Groningen, the Netherlands; k EBG MedAustron GmbH, Marie Curie-Straße 5, A-2700, Wiener Neustadt, Austria

**Keywords:** Pencil beam scanning, Small field dosimetry, Low-dose envelope, Double Gaussian beam model

## Abstract

**Purpose:**

The primary fluence of a proton pencil beam exiting the accelerator is enveloped by a region of secondaries, commonly called “spray”. Although small in magnitude, this spray may affect dose distributions in pencil beam scanning mode e.g., in the calculation of the small field output, if not modelled properly in a treatment planning system (TPS). The purpose of this study was to dosimetrically benchmark the Monte Carlo (MC) dose engine of the RayStation TPS (v.10A) in small proton fields and systematically compare single Gaussian (SG) and double Gaussian (DG) modeling of initial proton fluence providing a more accurate representation of the nozzle spray.

**Methods:**

The initial proton fluence distribution for SG/DG beam modeling was deduced from two-dimensional measurements in air with a scintillation screen with electronic readout. The DG model was either based on direct fits of the two Gaussians to the measured profiles, or by an iterative optimization procedure, which uses the measured profiles to mimic in-air scan-field factor (SF) measurements. To validate the DG beam models SFs, i.e. relative doses to a 10 × 10 cm^2^ field, were measured in water for three different initial proton energies (100MeV, 160MeV, 226.7MeV) and square field sizes from $1\;\times 1\;\;{cm}^{2}$ to $10\;\times 10\;\;{cm}^{2}$ using a small field ionization chamber (IBA CC01) and an IBA ProteusPlus system (universal nozzle). Furthermore, the dose to the center of spherical target volumes (diameters: 1cm to 10cm) was determined using the same small volume ionization chamber (IC). A comprehensive uncertainty analysis was performed, including estimates of influence factors typical for small field dosimetry deduced from a simple two-dimensional analytical model of the relative fluence distribution. Measurements were compared to the predictions of the RayStation TPS.

**Results:**

SFs deviated by more than 2% from TPS predictions in all fields $<4\;\times 4\;\;{cm}^{2}$ with a maximum deviation of 5.8% for SG modeling. In contrast, deviations were smaller than 2% for all field-sizes and proton energies when using the directly fitted DG model. The optimized DG model performed similarly except for slightly larger deviations in the $1\;\times 1\;\;{cm}^{2}$ scan-fields. The uncertainty estimates showed a significant impact of pencil beam size variations (±5%) resulting in up to 5.0% standard uncertainty. The point doses within spherical irradiation volumes deviated from calculations by up to 3.3% for the SG model and 2.0% for the DG model.

**Conclusion:**

Properly representing nozzle spray in RayStation’s MC-based dose engine using a DG beam model was found to reduce the deviation to measurements in small spherical targets to below 2%. A thorough uncertainty analysis shows a similar magnitude for the combined standard uncertainty of such measurements.

## 1 Introduction

Advances in proton beam delivery techniques and image guidance allow for irradiation of small target volumes such as boost volumes or small lesions in pediatric patients. This brings up new challenges for both treatment planning and clinical quality assurance. Determination of dose is conducted according to international standards, like the *IAEA Technical Reports Series No. 398* [1]. It states that the physical quantities that enter the beam quality correction factor $k_{Q,Q_{0}}$ are assumed to be reasonably independent of measuring depth and field size.

However, in treatment fields smaller than $4\;\times 4\;{cm}^{2}$ stopping-power ratios and perturbation factors might be impacted by field dimensions. In photon beam dosimetry this field size dependence has been extensively investigated and is accounted for by output correction factors that are numerically or experimentally assessed for each individual detector model [2]. The *IAEA Technical Reports Series No. 483* [2] provides a systematic and standardized procedure for dose measurements with high resolution detectors in small photon fields. In proton therapy no unified standard for small field dosimetry has been established. Although the general effects such as volume averaging are expected to be comparable in magnitude, an increased experimental uncertainty must be assigned to dose measurements in small proton fields as the current knowledge is somewhat limited.

More importantly, accurate clinical dose calculation is also challenging under small field conditions. Even when using Monte Carlo (MC)-based dose engines minor inaccuracies in the initial beam model can alter the lateral equilibrium in the center of the irradiation field. A proton pencil beam consists of a Gaussian-shaped core which is enveloped by a region of low fluence originating from large angle scattering. Following the terminology of Gottschalk et al. [3], charged secondaries originating from single large angle scattering in the medium are named halo whereas scattered radiation originating from the beamline components is referred to as nozzle spray. Several studies found that treatment planning and dose calculation can be affected when the low dose envelope is not properly represented in the algorithm’s beam model [4], [5], [6], [7], [8], [9], [10], [11], [12].

In modern treatment planning systems (TPS) the halo is inherently accounted for by MC-based dose algorithms, which explicitly model hadronic interaction processes on a microscopic level. The nozzle spray is not included in the dose computation procedure, but rather enters the calculation as the initial beams phase. Here, the impact of the fluence model depends strongly on the proton nozzle design. By approximating the initial beam profile with a single Gaussian (SG) function, as it is state of the art for most clinical dose algorithms, the off-axis proton fluence may be insufficiently modeled. This possibly influences calculated dose distributions, especially for small proton beams.

The exact shape of the pencil beam has been experimentally determined in various studies [5], [6], [7] and sophisticated models have been proposed and successfully tested [8], [9]. Ideally, the complete beam-transport through the pencil beam scanning nozzle would be simulated, intrinsically leading to an accurate prediction of the proton fluence [10], [11]. However, a weighted sum of two Gaussian functions can currently be considered the most practical approach regarding the balance between accuracy, computational speed and modelling efforts.

The aim of this work was to dosimetrically validate Monte Carlo dose calculations of the RayStation TPS (v. 10A; RaySearch Laboratories, Sweden) in small proton fields and systematically compare single Gaussian and double Gaussian (DG) modeling of the initial proton fluence. It was hypothesized that the use of a DG fluence model would result to a more accurate dose predictions in small proton fields due to a more precise representation of nozzle spray. For this purpose, two DG source models were created for the RayStation TPS from experimental data. To validate these models, MC-based dose calculations were conducted for monoenergetic square fields and energy modulated irradiation fields using both the SG and DG beam models. The resulting dose values were then compared to experimental data to systematically investigate the possible advantages of DG over SG source modeling within the MC-based algorithm.

## 2 Methods

All experiments were performed at the fixed beam line in the Proteus®PLUS facility (Ion Beam Applications Louvain-Neuve, Belgium) at the Westdeutsches Protonentherapiezentrum Essen (WPE). First, scan-field factors (SF) in monoenergetic square fields were measured to benchmark the dose algorithm under standardized conditions. In a second experimental series, spherical volumes of different diameters were irradiated to validate the dose calculation under clinically relevant conditions. A comprehensive uncertainty analysis was performed. The latter considered experimental influences associated with small field sizes i.e., positioning uncertainties, volume averaging and beam application uncertainties.

### 2.1 Source modelling in the TPS

Three different source models, an SG and two variants of a DG source model were commissioned in the RayStation TPS, to systematically compare the different approaches in terms of accuracy for dose predictions. RayStation’s MC dose engine uses these analytical models of the initial beam fluence to sample individual protons for MC dose calculation.

The single spot in-air dose profiles used in the beam modeling were recorded using the two-dimensional (2D) scintillator detector Lynx PT (*IBA Dosimetry, Germany*). Measurements were taken in five different distances to the isocenter (-25cm, -15cm, 0cm, +10cm, +30cm) for proton energies covering 100MeV to 226.7MeV in steps of 5MeV. To determine the profile tails down to a sub-percent level, the ‘pair magnification’ method, originally introduced by Lin et al. [4], was followed. In short, fluence distributions were acquired at two intensity levels by adjusting the proton machine monitor-units, thus leading to one image with a signal of 80% at maximum and one with a factor 10 higher, i.e. saturated. A post-processing routine was implemented in MATLAB (R2019a, MathWorks, Natick, US), scaling and merging the single measurements to a composite 2D image with signals down to 0.01% of the maximum.

The SG beam model was created with the RayStation auto modeling module. This procedure reconstructs the Fermi-Eyges (FE) parameters, a set of spatial-angular distribution moments defined at the isocenter plane: the angular variance, spatial-angular covariance, and spatial variance of the Gaussian distributed proton fluence Φ. These parameters were optimized by minimizing the difference between measured and calculated spot sizes (standard deviation) for each initial proton energy at various distances [12], [13], [14]. This optimization procedure in RayStation uses the average of one-dimensional profiles along the main axes of the 2D measurements.

Additionally, two different DG models were created, referred to as ‘*profile fit*’ and ‘*optimized fit*’ in the following. The two-dimensional models comprised a weighted sum of two 2D Gaussian functions with the radial distance r from the beam axis, with the individual weights $\omega_{1}$ and $\omega_{2}$ and the respective standard deviations $\sigma_{1}$ and $\sigma_{2}$.

(1) $$\Phi \left(r\right)=\omega_{1}e^{-r^{2}/2\;\sigma_{1}^{2}}+\omega_{2}e^{-r^{2}/2\;\sigma_{2}^{2}}$$

For the DG model ‘profile fit’ the parameters $\omega_{1}$ and $\omega_{2}$ and the standard deviations $\sigma_{1}$ and $\sigma_{2}$ were determined using a least-squares fit in MATLAB (R2019a) for each dataset per energy and distance. The fit was performed on the 2D images in linear space down to the aforementioned intensity level of 0.01%. The underlying fit model was constrained to a symmetric solution along the main axes, i.e. a possible spot-shape rotation and asymmetry was not considered. Pursuing the approach of the built-in auto modeling in RayStation, the FE-parameters were then deduced by minimizing the difference of measured vs. calculated spot size by solving the linear-quadratic FE transport-function, independently for the primary and secondary Gaussian. Finally, the parameters $\omega_{1}$ and $\omega_{2}$ were taken as an average over all five distances to isocenter for each energy.

The parametrization of the DG model ‘optimized’ used the same input data as the ‘profile fit’ model, but the fit was not performed using the measured single spot profiles directly. Instead, the single spot profiles were used to simulate the cross-section profiles of scanned quadratic fields of varying size. The simulation was realized by superpositioning of the measured single spot profiles organized in a regular spot pattern that corresponded to the simulated scanned field. In the extraction of the phase space parameters, the modelled profiles were calculated using error functions taking the standard deviations $\sigma_{1}$ and $\sigma_{2}$, as well as the parameters $\omega_{1}$ and $\omega_{2}$ in equation (1) as input [15]. For each energy, the relative weights, and the phase space parameters of the two Gaussians at isocenter were determined in an optimization including fits to the simulated profiles of all field sizes and all distances from isocenter simultaneously. In each optimization iteration, the modelled spot size at each distance from isocenter was computed from the phase space parameters at isocenter by FE transport assuming vacuum [16]. The potential advantage of this method is that the fit is performed on data that is closer to how the model will be used, and that one can focus on a certain field size range for the model. For the ‘optimized’ DG model of this study, field sizes between $1\;\times 1\;\;{cm}^{2}$ and $10\;\times 10\;{cm}^{2}$ were used, and the profiles at isocenter were weighted higher in the optimization than the upstream/downstream profiles.

The transport mechanics and physics models used in the range shifters and in the phantom were identical for all beam models, i.e. particle transport starts with propagated FE-parameters at the range shifter entrance [17]. The RayStation MC-algorithm has been shown to sufficiently represent the halo in medium [14]. Properties of range shifters in terms of thickness and material were provided by the user as part of the beam-model. The derived energy-dependent FE-parameter tabulations were finally loaded into separate RayStation beam models. We note that there is no field size dependent output correction factor in the RayStation beam model.

### 2.2 Dosimetric Validation

The dosimetric validation consisted of two experimental series with varying complexity. The performance of dose calculations with the SG and DG beam models was compared to dosimetric measurements. At first SFs i.e., the central dose in monoenergetic square fields of different sizes relative to the central dose of a $10\;\times 10\;{cm}^{2}$ scan-field, were acquired. Under these conditions, the influence of proton energy and field size on the agreement between calculated and measured dose was investigated.

In the second experimental series, modulated proton fields covering spherical volumes were studied. The depth and diameter of the spheres were varied to cover possible clinical cases in small field proton therapy.

All fields were applied using the pencil beam scanning mode without any additional collimation. All dose distributions were calculated using the RayStation 10A clinical Monte Carlo algorithm v5.0 on a $1\;\times 1\;\times 1\;{mm}^{3}$ voxel grid ensuring a mean relative statistical uncertainty of 0.2% or less in the relevant parts of the field.

#### 2.2.1 Scan-field factors

Monoenergetic square fields with eight scan-field sizes ranging from $1\;\times 1\;{cm}^{2}$ to $10\;\times 10\;cm^{2}$ and proton energies of 100MeV, 160MeV and 226.7MeV were studied. The energies were selected to cover the full commissioned range at WPE. The approach of Shen et al. [18] was followed to measure all eight scan-field sizes within a single beam request. For this purpose, the spot pattern of a $10\;\times 10\;\;{cm}^{2}$ reference field was divided into eight concentrical bands that were irradiated and measured individually. Beam pauses were induced after every portion of the field to read out the measured charge. By summing up all readings up to a certain scan-field size, the charge per resulting field size was obtained.

Here and in the following, the term ‘scan-field size’ refers to the spatial dimensions of the nominal spot positions. The spot pattern for the square fields was created by manually editing the machine files using a 2.5mm spot spacing and constant spot weights. Spot sigma was 8.1mm, 5.1mm and 3.2mm at the iso plane for 100MeV, 160MeV and 226.7MeV, respectively.

Measurements were performed in the plateau region of the Bragg peak at 3cm water-equivalent depth (WED) in the center of the square fields using the small volume ($1\;\cdot 10^{-2}cm^{3}$) IC CC01 (SN: 8972, *IBA Dosimetry, Germany*), a DOSE-1 reference class electrometer (*IBA Dosimetry, Germany*) and a type 41023 water phantom (*PTW-Freiburg, Germany*) with a custom-made chamber holder. The detector was irradiated radially and the shift of the effective point of measurement (EPOM) from the IC’s center was accounted for by 0.75 times the inner radius of the individual IC (DIN 6801-1). The entrance window of the phantom was aligned perpendicular to the beam central axis using the X-ray imaging system. The isocenter was positioned in 3cm WED and alignments of the detector and the phantom were verified by X-ray imaging.

#### 2.2.2 Small irradiated volumes

Spherical target volumes with diameters of 1cm, 2cm, 4cm, 6cm and 10cm were irradiated in WED of 5cm, 10cm, 20cm and 25cm. The isocenter was positioned at the point of measurement in the center of the spherical target volumes.

The irradiation fields were optimized within RayStation leading to a uniform RBE-weighted dose of 180cGy within the target structure. Here, 98% of the respective target volume had to receive at least 175cGy of RBE-weighted dose. Additionally, a dose fall-off objective from 180cGy to 90cGy RBE-weighted dose within 1cm was defined. The default spot and layer spacing of RayStation (“automatic with scale 1”) was applied, i.e. spot spacing of 1.06 times the average projected sigma and layer spacing that corresponds to an intersection at the 80% dose level of the next distal Bragg peak.

Range shifters (polymethyl methacrylate) were used to cover shallow targets: 74mm water equivalent thickness (WET) for 5cm depth and diameters of 10cm, 6cm and 4cm. 51mm WET for 5cm depth and diameters of 2cm and 1cm. And 25mm WET for 10cm WED and diameters of 10cm and 6cm.

Experiments employed the IBA CC01, the DOSE-1 electrometer and the type 41023 water phantom to measure dose to water in the center of the spheres. The detector position in the corresponding depth was validated by means of X-ray imaging. The dose to water was evaluated according to DIN6801-1 [19] including correction factors for air density, polarity effect and detector positioning (EPOM). Ion recombination correction was evaluated for proton energies of 100MeV, 160MeV and 226.7MeV using the two-voltage method and $k_{s}$ taken as a mean value over all energies [20]. The beam quality correction factor $k_{Q,Q_{0}}=1.060$ was assessed from a cross calibration with the Exradin T1. Reference dosimetry with the T1 IC was established at WPE [21], [22] and literature values for the T1’s $k_{Q,Q_{0}}$ are in good agreement with water calorimetry [23].

#### 2.2.3 Uncertainty analysis and numerical estimations

Experimental uncertainties of both measurement series were evaluated as type B standard uncertainties according to the Guide to the Expression of Uncertainty in Measurement (GUM) [24] and compiled in an uncertainty budget.

The uncertainty of the calibration and calibration drift of both the detector and the electrometer were taken from calibration certificates and manufacturer’s manual. If standard uncertainty was not explicitly stated, it was estimated by $u_{c}=M/\sqrt{3}$ assuming a constant probability density with M being the maximum given deviation [1], [24].

The uncertainty resulting from disregarding the correction factors for humidity $k_{h}$ and temperature $k_{T}$ were acquired according to DIN 6801-1 [19]. The experimental uncertainty of measuring in fields smaller than the reference field size, which originates from changes in Bragg-Gray cavity conditions, was estimated based on publications by Gomà et al. and Kretschmer et al. [25], [26]. Goma et al. investigated Spencer–Attix water/medium stopping-power ratios in single proton pencil beams, while Kretschmer et al. simulated perturbation correction factors for cylindrical ICs in monoenergetic proton beams. Maximum variations of these quantities were used to estimate a type B uncertainty for measurements in smaller than reference field sizes. Again, constant probability density was assumed [24].

Since the reproducibility of field application and the correction factors for air mass $k_{\rho }$, ion recombination $k_{s}$, polarity effect $k_{p}$ and beam quality $k_{Q,R}$ were experimentally assessed, an uncertainty budget was compiled for each of these quantities including some of the above-mentioned uncertainties.

Additionally, four aspects that may have affected the measurement in the monoenergetic square fields were studied: detector positioning errors, impact of volume effect, pencil beam positioning and variation in pencil beam size. For this purpose, a two-dimensional numerical model of the dose distribution was created in MATLAB R2020b. In this model, single pencil beam positions of square monoenergetic fields (2.5mm spot spacing) were defined by a two-dimensional Dirac comb and convolved with a two-dimensional Gaussian function, representing the proton fluence of a single pencil beam. This operation resulted in a high-resolution (0.1mm) image of the two-dimensional distribution in the experimental square fields. Under the assumption that proton fluence is proportional to dose in the lateral plane, the calculated distribution was used for estimating the measurement uncertainty. The σ (standard deviation) of the 2D-Gaussians was chosen to represent different proton beam energies in accordance with spot profiles measured in air. The additional broadening due to scattering in 3cm WED was not considered. Detector specific SF measurements were mimicked by determining mean values over a central square area, representing the detectors cross section, and calculating dose ratios to a $10\;\times 10\;{cm}^{2}$ field. The CC01’s cross sectional area was estimated to be $7.2\;mm^{2}$ by assuming a rectangular shape with side lengths of the nominal chamber diameter (2mm) and chamber length (3.6mm) [1]. By changing the spot pattern and σ, a variety of field sizes and proton energies were modeled. The influence of detector positioning errors, volume averaging effect, pencil beam positioning errors and variation in pencil beam size on the SF measurements were calculated as follows.

*Detector’s volume averaging*: The relative deviation between the mean value over the detector cross-section and central pixel values ($0.1\;\times 0.1\;{mm}^{2}$) were considered as limit of a uniformly distributed probability density. The experimental standard uncertainty resulting from disregarding the volume averaging effect was calculated based on this detector-specific value. Since the volume effect in proton radiation fields is mainly due to volume averaging rather than density perturbations by non-water equivalent detector components [27], this calculated uncertainty was considered a reasonable estimate for neglecting the volume effect.

*Detector positioning errors*: Uncertainties were estimated by horizontally and vertically shifting the detector area by a normally distributed random number with standard deviation of 0.5mm. These shifts corresponded to treatment table positioning uncertainties of ±0.1mm and the collinearity between the X-ray system and proton beam axis of <0.5mm. SF calculations using the MATLAB model were repeated multiple times for each field size with the number of repetitions increasing towards smaller field sizes. The experimental uncertainty due to detector positioning errors was taken to be the standard deviation for every set of calculations.

*Pencil beam positioning* errors: Experimental uncertainties were estimated by randomly shifting spot positions (standard deviation: 0.25mm) and determining the standard deviation of repeated SF calculations per field size. A similar approach has recently been published by Medin et al. [28].

*Spot size deviations: S*pot sigma in the MATLAB model was changed by ±5%. Maximum deviations of the numerically generated SFs were taken as a limit of a uniformly distributed probability density to calculate the experimental standard uncertainty. Deviations of spot sizes from TPS commissioning values were tested regularly in clinical quality assurance procedures and are typically ≤5%.

## 3 Results

### 3.1 Source modelling in the TPS

**Figure 1:**
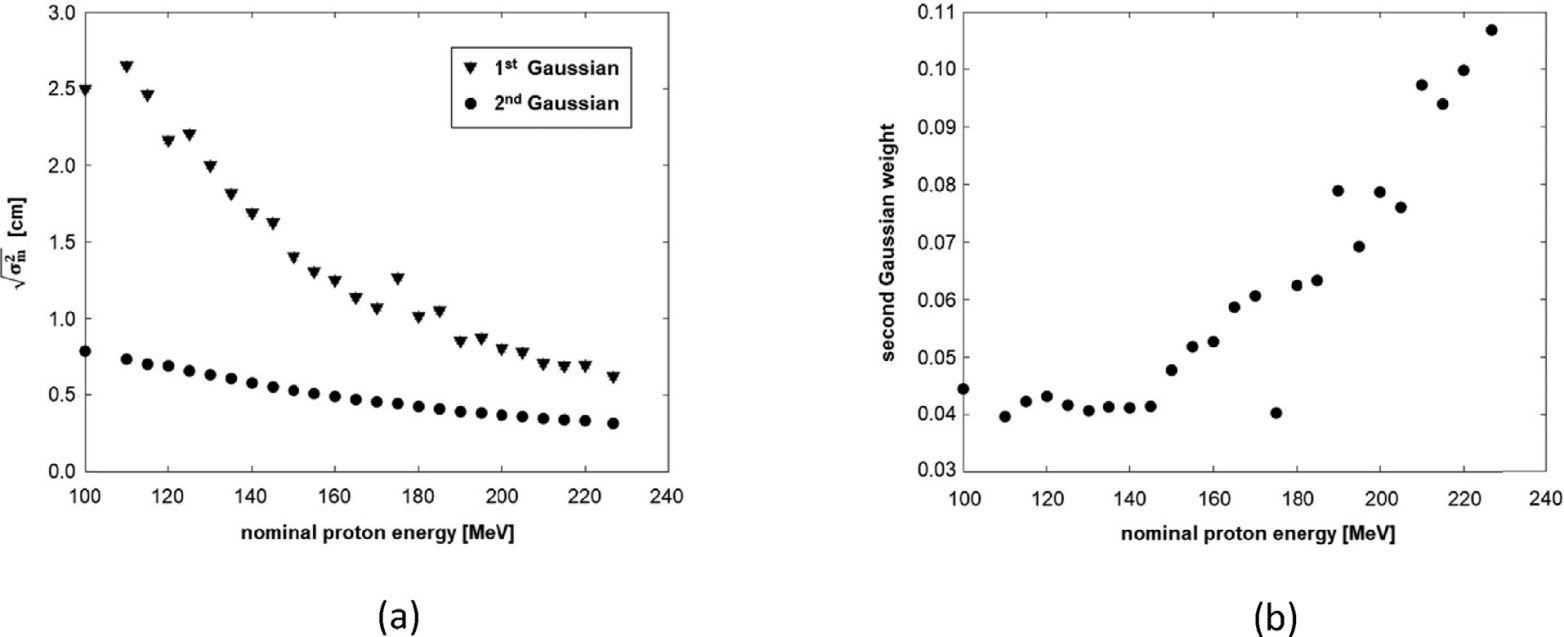
Optimized DG beam model: (a) sigma of first and second Gaussian depending on proton energy; (b) relative weight of second Gaussian depending on proton energy.

Fig. 1 displays the standard deviation $\sigma_{1}$ and $\sigma_{2}$ and the parameters $\omega_{1}$ and $\omega_{2}$ for the optimized DG source model depending on proton energy. Both standard deviations increased with decreasing proton energy. The relative difference between $\sigma_{1}$ and $\sigma_{2}$ became considerably larger at low proton energies, while the relative weight of the second Gaussian substantially increased towards higher energies (see Fig. 1).

### 3.2 Dosimetric validation

#### 3.2.1 Scan-field factors

**Figure 2:**
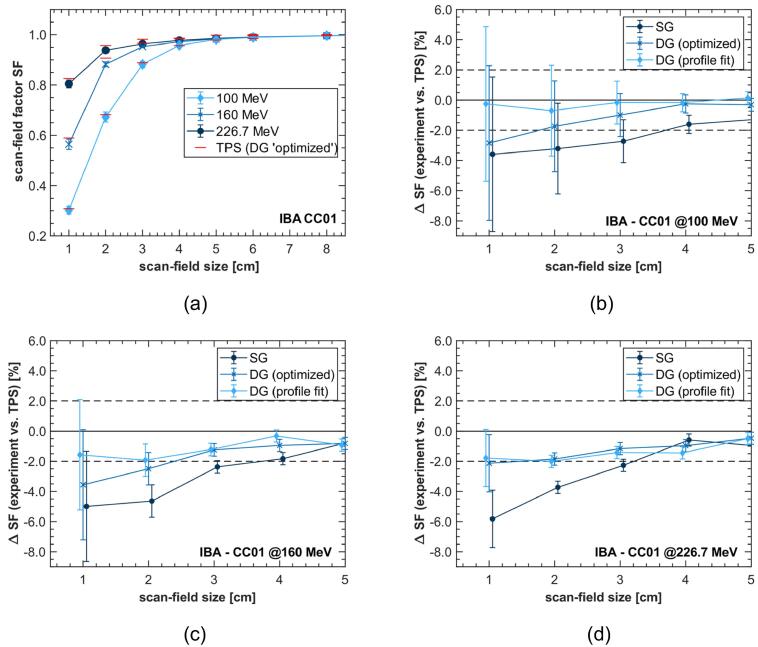
(a) Scan-field factors (SFs) measured with CC01 chamber at three different proton energies depending on scan-field size; (b)-(d) deviation ΔSF between measured and calculated SFs for different source models; scan-field size is given as side length of square spot pattern; lines between data points are intended to guide the eye and do not represent continuous data.

Fig. 2 (a) displays the scan-field factors measured for the three proton energies. The measured values decreased towards smaller scan-field sizes, i.e. following the general behavior known from small field photon and passive proton beam dosimetry [29]. This characteristic field size dependency was more pronounced at lower proton energies, since spot size typically increases with decreasing energy [30]. A SF as low as 0.3 was measured for the $1\;\times 1\;\;{cm}^{2}$ field at 100MeV.

The relative deviations of the experimentally determined SFs from the values calculated with the different source models are shown in Fig. 2 (b)–(d). Small field SFs calculated with the DG source models showed closer agreement to experimental values than those calculated with the SG source model. When using the SG model in scan-field sizes $<4\;\times 4\;{cm}^{2}$ the relative deviations exceeded 2% at all energies, with a maximum error of 5.6%. In contrast, all deviations were within 2% (AAPM tolerance limit for SF calculation with MC-based TPS in photon therapy [31]) for the ‘profile fit’ DG model. The relative deviations for the optimized DG model in the $1\;\times 1\;cm^{2}$ scan-field were slightly larger, with 2.8%, 3.6% and 2.1% at 100MeV, 160MeV and 226.7MeV, respectively. This could likely be improved by a higher weight of the smallest field size in the beam modeling (see Section 2.1).

#### 3.2.2 Small irradiated volumes

**Figure 3:**
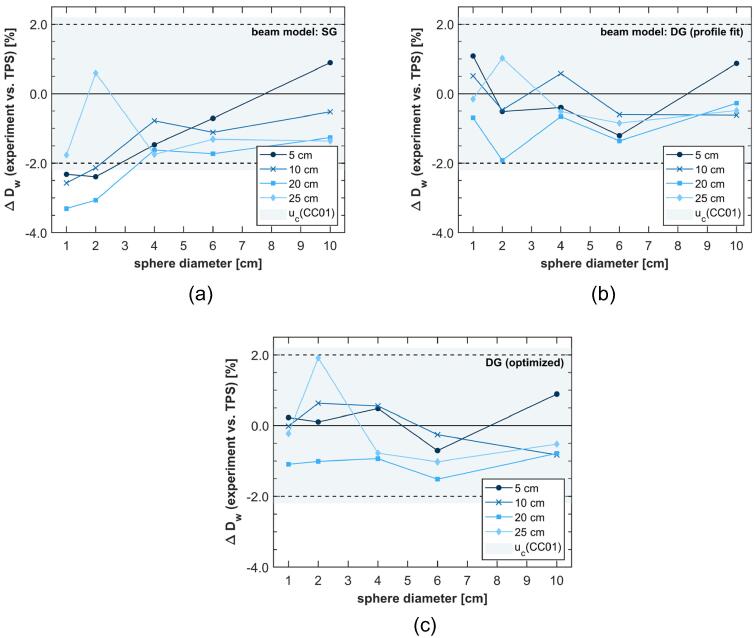
Deviation $\Delta D_{w}$ between measured (CC01) and calculated dose to spherical target volumes; depth of sphere as parameter; (a) SG source model; (b) DG source model fitted to spot profiles; (c) DG beam model optimized by SF calculations; light blue shading represents the standard uncertainty of CC01 measurements; lines between data points are intended to guide the eye and do not represent continuous data.

Fig. 3 illustrates the deviation $\Delta D_{w}={(D}_{\exp}-D_{\mathrm{TPS}})/D_{\mathrm{TPS}}$ between measured dose $D_{\exp}$ and calculated dose $D_{\mathrm{TPS}}$ in the center of spherical target volumes as a function of the sphere diameter for the different sphere depths. Panels (a) to (c) display the data for each of the three different beam models. Note that the shaded area represents the standard uncertainty of CC01 measurements (see Section 3.2.3).

For the SG model, $\Delta D_{w}$ increased systematically with decreasing sphere diameter. Like in the SF series, the deviations exceeded 2% below target sizes of 4cm for all but the shallowest depth. For the DG models, $\Delta D_{w}$ appeared more random and stayed within 2% as well as within the CC01’s standard uncertainty for all field-sizes. No clear difference was seen between the two types of DG models.

In large proton fields (>4cm), in which the measured dose is expected to agree well with the TPS-calculated values, a constant dose offset of about 0.5% to 1% could be observed for water depths of 10cm to 25cm and for all source models, which was however well within the estimated uncertainty (see Fig. 3).

#### 3.2.3 Uncertainty analysis and numerical estimations

Table 1 and Table 2 present the uncertainty budgets for measuring the SF and the dose to spherical volume, respectively. The experimental uncertainty for smaller than reference field sizes was estimated to be 0.4%.

**Table 1:** Uncertainty budget for SF measurements using different detectors including standard uncertainties u.

No.	influence on measurement	u [%]
1	dosimeter reading	
1.1	uncertainty of charge measurement	0.1
1.2	neglection of volume averaging*	2
1.3	detector positioning*	0.7
2	beam application	
2.1	spot size variability*	5
2.2	pencil beam positioning*	1
2.3	reproducibility	0.1
3	beam quality	
3.1	scan-field size	0.4
4	combined standard uncertainty**	0.4

* Estimates from numerical model; maximum values regarding detector size, scan-field size and proton energy are given.

** Combined standard uncertainty is given without numerically estimated uncertainties (neglection of volume averaging, detector positioning, spot size variability, pencil beam positioning) because these depend on energy, field size and detector model.

**Table 2:** Uncertainty budget for measuring dose to spherical target volumes using the ionization chamber CC01 including standard uncertainties u.

No.	influence on measurement	u [%]
1	calibration factor	
1.1	uncertainty of detector calibration	0.5
1.2	drift of calibration	0.3
2	dosimeter reading	
2.1	uncertainty of electrometer calibration	0.1
2.2	drift of electrometer calibration	0.06
2.3	uncertainty of charge measurement	0.1
2.4	neglection of volume averaging*	0.2
2.5	detector positioning*	0.1
2.6	beam application reproducibility	0.1
3	beam quality correction	
3.1	$k_{\mathrm{TP}}$	0.3
3.2	$k_{h}$(humidity)**	0.06
3.3	$k_{s}$	0.2
3.4	$k_{p}$	0.6
3.5	$k_{T}$(temperature effects other than air density)**	0.06
3.6	$k_{Q_{\mathrm{ref}},Q_{0}}^{f_{\mathrm{ref}}}$(cross calibration)	1.9
3.7	$k_{Q_{\mathrm{clin}},Q_{\mathrm{ref}}}^{{f_{\mathrm{clin}},f}_{\mathrm{ref}}}$(correction for field size)**	0.4
4	combined standard uncertainty	2.2

* Maximum values regarding beam size are given.

** Correction factors according to DIN 6801-1; uncertainty resulting from disregarding correction.

The experimental uncertainty for the dose measurements in spherical fields was dominated by the $k_{Q,Q_{0}}$ cross calibration uncertainty of 1.9%. This uncertainty originated from the experimental uncertainty of charge measurements, the calibration uncertainty of both ICs, the uncertainty of beam quality correction $k_{Q,Q_{0}}$ (estimate taken from TRS-398, table 10.IV) and the uncertainty of the correction factor $k_{\mathrm{TP}}$.

**Figure 4:**
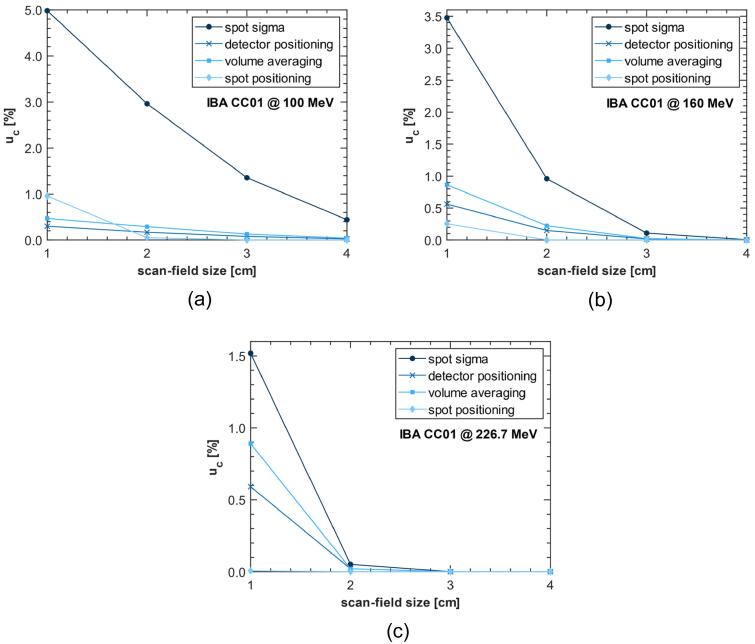
Experimental standard uncertainties due to disregarding volume averaging, spot sigma variations of 5%, spot positioning errors (normally distributed with sigma of 0.25mm) and detector positioning errors (normally distributed with sigma of 0.5mm); values were analytically estimated using two-dimensional numerical calculations; scan-field size is given as side length of square spot pattern; lines between data points are intended to guide the eye and do not represent continuous data.

Fig. 4 illustrates the numerically determined standard uncertainties from detector positioning errors, volume averaging effect, pencil beam positioning and variation in pencil beam size (see Section 2.2.3). In general, uncertainties gradually increased with decreasing scan-field sizes below $4\;\times 4\;{cm}^{2}$.

As expected, the calculations showed that disregarding volume averaging in the measurement process results in significant inaccuracies in small proton beams. Uncertainties increased with decreasing scan-field size reaching their maximum in the $1\;\times 1\;cm^{2}$ field at maximum proton energy (see Fig. 4).

The numerical model showed that uncertainties due to spot sigma variations of ±5% have by far the largest impact on combined standard uncertainty, covering up most of the remaining influence factors. A standard uncertainty of up to 5.0% was assessed for proton energies of 100MeV (see Fig. 4a). The measurement uncertainty due to volume averaging was substantially lower and was less than 1% for all proton energies. In the numerical analysis lower proton energies generally resulted in higher standard uncertainties, except for volume averaging and detector positioning in the smallest scan-field size of $1\;\times 1\;{cm}^{2}$.

## 4 Discussion

### 4.1 Scan-field factors

The deviations between SF measurements and TPS calculations were affected by the type of beam model as well as detector effects of the IC CC01 like volume averaging and perturbations and other experimental uncertainties. This impedes distinguishing how these quantities affect the agreement of dose predictions and experimental data.

Considering the small field validation limit of RayStation MC of $4\;\times 4\;{cm}^{2}$ and the AAPM tolerance limit for SF calculation with MC-based TPS in photon therapy (2%), experiments showed highly satisfying agreements with calculations done using DG beam model, even for field sizes $<4\;\times 4\;{cm}^{2}\;$[17], [31]. Under these conditions, 77.8% of the measured values showed deviations ≤2%. Differences between SG and DG beam models were most pronounced when using 226.7MeV proton energies to irradiate fields $<4\;\times 4\;{cm}^{2}$ (see Fig. 2d). Despite higher proton energies generating less nozzle spray and smaller second Gaussian sigma, the relative weight of the second Gaussian becomes considerably larger resulting in improved agreement to experimental data (see Fig. 1).

In analytical dose engines, the impact of neglecting halo and nozzle spray in beam modeling has already been extensively investigated. In a work similar to the present study, Zhu et al. [32] compared SFs calculated with Eclipse’s pencil beam algorithm with experiments done on a first generation PBS nozzle (Hitachi). They found that disagreements between calculated and measured SFs peaked towards the smallest field size of $2\;\times 2\;{cm}^{2}$ with deviations of up to 15% when using a single Gaussian beam model. With the addition of a second Gaussian in the fluence model, the error could be kept below 2% for most energies and depths. It is important to note that Zhu et al. [32] and Shen et al. [18] manually adjusted the weight and size of the second Gaussian to compensate the inadequate handling of the halo produced within the water phantom. In the work of Zhu et al. [32] the field size error was as large as 10% when the second Gaussian model was fitted directly to measured in-air spot profiles. Even though the resulting agreements for the DG model are of the same magnitude as those presented in this paper, results are not comparable, because here DG beam modeling is based on in-air measurements only. Further, the deviation of pencil beam profiles from a single Gaussian distribution is more pronounced for the Hitachi nozzle used by Zhu et al. [32] and Shen et al. [18] compared to the IBA nozzle used in the present study.

In another previous experiment Harms et al. [30] used a clinical MC-based TPS with SG source model to calculate SFs for comparison with IC measurements. At low proton energies and small field sizes, substantial disagreements were detected, indicating a poorly modeled nozzle spray. The authors argued that a DG representation of the initial beams phase might improve the observed deviations. The results of the present study support the hypothesis that DG beam modeling can improve agreement to experimental values even for MC-based TPS.

The results of SF measurements in small proton fields ($<4\;\times 4\;{cm}^{2}$) show that the agreement between experimental dose values and MC-based dose calculations can be improved by utilizing DG modeling for a more accurate representation of the nozzle spray, keeping the dose error below 2% even for targets as small as 1cm. In the center of broad irradiation fields, differences in the SG and DG beam model are canceled out by neighboring pencil beams. For small field sizes, the DG beam model yields lower doses on the central axis because more proton fluence is deposited outside the irradiation field compared to the SG beam model.

### 4.2 Small irradiated volumes

Spherical target volumes with diameters starting at 1cm served as substitute for clinically relevant targets while also allowing for a systematic evaluation of the field size dependence. Deviations ${\Delta D}_{w}$ between CC01 measurements and calculations were within 2% for DG beam modeling, which is the tolerance level for MC-based patient dose calculations in photon therapy defined in the AAPM report 157 [31]. Additionally, the deviations ${\Delta D}_{w}$ remained smaller than the combined standard uncertainty when using the DG source model (see Fig. 3). Considering that, results demonstrate that reliable dose predictions can be made even below the smallest field size (4cm) validated in the RayStation [17]. Especially in small fields (≤2cm) DG beam modeling improved agreement compared to SG beam modeling (see Fig. 3).

The results of CC01 measurements demonstrate that treatment planning, field application and quality assurance procedures in our facility are well suited for the treatment of the smallest clinically relevant lesions, with planning target volumes starting at a diameter of 1cm. The DG beam model provides better results in these small proton fields, that are highly challenging in clinical application.

There is a considerable contribution to CC01 measurement uncertainty from cross calibration. A more direct comparison of the models would be possible if the same IC was used for absolute dose calibration of the TPS beam model and the determination of the dose in the center of the spherical targets. In this case, the calibration would be purely based on detector reading. One could also envision to use MC based $k_{Q,Q_{0}}$ and corresponding uncertainty estimations. Generally, these $k_{Q,Q_{0}}$ factors carry a lower uncertainty [33].

In an experiment similar to the present study, Würl et al. [34] irradiated spherical target volumes at 15cm water depth. Here, the diameter of the fields was varied between 2cm and 10cm. Maximum deviations of 0.8% were observed when comparing the experimental results to dose values calculated with a commercial pencil beam algorithm [34]. Considering the large differences between experimental and TPS-calculated SFs reported in the same paper it remains unclear if the dose calculation for the spherical targets was internally corrected by output factors and/or further validation data. Therefore, the reported agreements between calculated and measured dose, that are of similar magnitude as those presented in Fig. 3 (b) and (c), might not be comparable due to differences in the dose calculation procedure. All dose calculations presented here utilize source models created on experimental, in-air data only and are not subsequently corrected, e.g. for field size. The 0.5% to 1% offset observed for larger proton fields (see Fig. 3) could have been caused by calibration drift of the detector, which is accounted for in the uncertainty budget.

**Figure 5:**
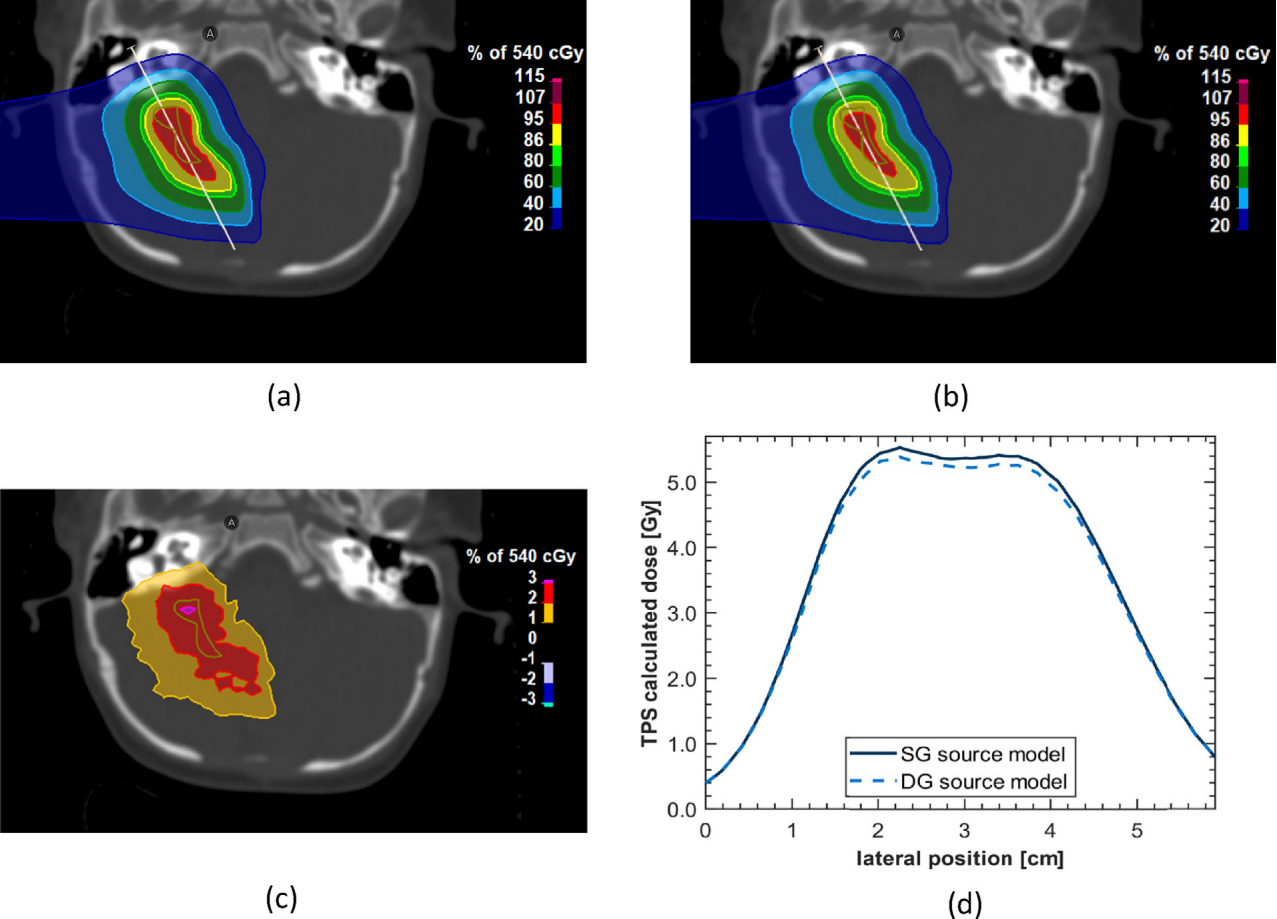
Comparison of a dose distribution in head and neck tumor patient calculated with RayStation MC dose engine using SG model (a) vs. DG source model (b). The original plan was optimized with the SG model.; (c) spatial dose difference in percent (SG-DG); (d) line doses along the indicated in panel (a).

Fig. 5 exemplarily illustrates the impact of using a DG source model as compared to an SG source model for dose calculations with the RayStation MC algorithm in a two-field, pediatric head and neck tumor patient. Dose differences of up to 3% can be observed in this relatively small treatment field demonstrating the clinical relevance. Further analysis is beyond the scope of this study and more research is needed to assess the clinical impact.

### 4.3 Uncertainty analysis and numerical estimations

The numerical uncertainty estimates showed increasing contributions to the measurement uncertainty for scan-field sizes < $4\;\times 4\;{cm}^{2}$. This matches the small field limit mentioned in photon dosimetry [35].

Variations in spot size of ±5% were found to have the largest impact on combined standard uncertainty. Even though Rana et al. [36] demonstrated that the long-term fluctuation in spot-sizes for an IBA proton therapy system was less than 5% (especially for lower energies) and day-to-day spot size variations in our facility stay well within 5%, this tolerance was used in the numerical model as a conservative estimate. In literature, changes of 10% to 50% are typically assumed to have a negligible effect on clinical dose distributions [37], [38], [39], [40], [41]. The results illustrated in Fig. 4 indicate that variations of ±5% can already have a notable effect on dosimetry in small proton fields.

Numerically estimated uncertainties were generally lower for higher proton energies. This can be attributed to the fact that the spots are smaller at higher energies, resulting in a more homogeneous dose in the field’s center (see Fig. 4). A reversal of this trend was observed in the $1\;\times 1\;{cm}^{2}$ irradiation field for uncertainties that result from detector positioning errors and disregarding volume averaging. The $1\;\times 1\;{cm}^{2}$ irradiation field is generated of only 25 pencil beams so that the field mainly consists of the field’s edge. Here, lower proton energies lead to broader fields inside the detector area so that uncertainties due to positioning errors and volume averaging are less pronounced.

The standard uncertainty due to the neglected volume averaging effect was calculated to be of the same magnitude as correction factors for non-reference field-size known from photon dosimetry [42]. This supports the validity of the numerical approach, since variances of similar magnitude are to be expected in small field proton dosimetry [43] and the volume effect in proton radiation is mainly caused by volume averaging [27].

## 5 Conclusion

The present study investigated the impact of double Gaussian beam modeling on proton dose calculations done with RayStation 10. While previous works focused on DG beam modeling in analytical dose algorithms taking the field size measurement results as input, this study investigated DG beam modeling based on in-air measurements only, in a commercial MC-based dose engine for the first time. Results show that DG beam modeling improved the agreement between TPS and experimental data in small proton fields ($<4\;\times 4\;{cm}^{2}$), indicating that MC-based dose engines can benefit from an accurate representation of the nozzle spray in the initial beam phase modeling. Since the DG model was based on in-air measurements only, these results also show that the modeling of, mainly, the non-elastic nuclear scattering in the RayStation 10 MC dose engine is sufficiently accurate to predict dose to within 2% accuracy for target sizes down to 1cm at depths ranging from 5cm to 25cm. When irradiating spherical target volumes with diameters corresponding to the smallest clinically relevant structures, disagreements between DG-based calculations and dose measurements were within the AAPM limit for patient dose calculations in photon therapy (2%). These dosimetric measurements are well supported by a comprehensive analysis of experimental uncertainties. Therein, numerical estimations showed that variations in spot size of ±5% could significantly impact dosimetry of small proton fields.

In conclusion, findings indicate that DG beam modeling, available for MC dose calculation in RayStation, is not only practicable in clinical applications but can also significantly improve dose calculations in small proton irradiation fields.

## Author contribution

All the above listed authors directly contributed to this work (design, acquisition, analysis, interpretation), drafted/revised the text critically and approved the final version to be published. All authors agreed to be accountable for all aspects of the work ensuring that questions related to the accuracy or integrity of any part of the work are appropriately investigated and resolved. F. Kugel and J. Wulff developed the design for this study. The data acquisition was performed by F. Kugel, J. Wulff, C. Bäumer, J. Kretschmer, L. Brodbek, C. Behrends, N. Verbeek. The experimental results were analyzed by F. Kugel, J. Wulff and M. Janson and interpreted by F. Kugel, J. Wulff, C. Bäumer, J. Kretschmer, L. Brodbek, C. Behrends, N. Verbeek, H.K. Looe, B. Poppe and B. Timmermann. The script was drafted by F. Kugel, J. Wulff and M. Janson.

## Declaration of Competing Interest

The authors declare the following financial interests/personal relationships which may be considered as potential competing interests: Martin Janson is employed by RaySearch Laboratories AB. The remaining authors have no conflict of interest to disclose.
